# Supplementary material for: Development of a Novel Blood‐Based Assay for Brain‐Derived Tau and Its Validation in Traumatic Brain Injury
Source: J Neurochem. 2026 Jul 18;170(7):e70518. doi: 10.1111/jnc.70518 (PMC13379723; doi:10.1111/jnc.70518)
Supplement: Supplementary file 1 — Table S1: LLOQ AEB values. Table S2: Intra‐ and inter‐plate coefficients of variation. Table S3: Full statistical report on TBI severity, outcome and survivability. Table S4: Unadjusted model of performative prediction of Pitt‐BD‐tau in predicting TBI. Figure S1: Four‐parameter logistic (4PL) regression calibration curve on a log–log scale. The curve was generated with nine non‐zero calibrators that are used with the Pitt‐BD‐tau assay. [file JNC-170-e70518-s001.pdf]

Development of a Novel Blood-Based Assay for Brain-Derived Tau and Its Validation in Traumatic Brain Injury

Wasiu G. Balogun[a,b,c], Xuemei Zeng[a,b,c], Michel N. Nafash[a,b,c], Anuradha Sehrawat[a,b,c], Ruyu Shi[a,b,c], Sarah E. Svirsky[d], David O Okonkwo[d], Ava M. Puccio[d], Thomas K. Karikari[a,b,c]

[a]Department of Psychiatry, University of Pittsburgh, Pittsburgh, PA, USA

[b]Alzheimer's Disease Research Center, University of Pittsburgh, Pittsburgh PA, USA

[c]Biofluid Biomarker Laboratory, Western Psychiatric Hospital, University of Pittsburgh Medical Center, Pittsburgh PA, USA

[d]Department of Neurological Surgery, University of Pittsburgh, Pittsburgh, PA, USA.

Correspondence: Dr Thomas K. Karikari, Department of Psychiatry, University of Pittsburgh School of Medicine, Pittsburgh, PA, USA. Email: Karikari@pitt.edu; Karikaritk@upmc.edu

Supplementary Tables

Table S1. LLOQ AEB values

Replicate	AEB value
LLOQ1	0.127457
LLOQ2	0.132806
LLOQ3	0.127172
LLOQ4	0.123612
LLOQ5	0.12205
LLOQ6	0.120689
LLOQ7	0.123146
LLOQ8	0.129976
LLOQ9	0.132702
LLOQ10	0.132628
LLOQ11	0.130304
LLOQ12	0.127551
LLOQ13	0.119809
LLOQ14	0.116182
LLOQ15	0.121315
LLOQ16	0.120281

Table S2. Intra- and inter-plate coefficients of variation

Sample	Intra-plate CV (%)	Inter-plate CV (%)
QCL	5.482	10.992
QCLMG	12.177	19.010
QCSERUM1	8.180	15.052
QCSERUM2	5.147	9.679
QCSERUM3	6.993	10.262
QCSERUM4	5.964	8.039

Table S3. Full statistical report on TBI severity, outcome and survivability

**Figure 3A**	**Group (Control, Chronic-mixed, and Severe acute) differences in 2-step BD-tau**					
	**Kruskal-Wallis** H = 42.518, df = 2, p = 5.85E-10					
	**Dunn's post-hoc**	**group1**	**group2**	**z**	**p_raw**	**p_bonf**
		Control	Chronic-mixed	0.879	0.380	1.000
		Control	Severe-acute	4.830	1.37E-06	4.12E-06
		Chronic-mixed	Severe-acute	5.520	3.36E-08	1.01E-07
	**ANCOVA adjusted for sex and age (using log10-transformation on biomarkers):** F(2,57) = 45.02, p = 1.86E-12					
	contrast	estimate	SE	df	t.ratio	p.value
	Control - (Chronic-mixed)	-0.041	0.118	57	-0.346	0.730
	Control - (Severe-acute)	-0.711	0.113	57	-6.306	6.77E-08
	(Chronic-mixed) - (Severe-acute)	-0.670	0.080	57	-8.379	4.85E-11
**Figure 3B**	**Group (Control, Chronic-mixed, and Severe acute) differences in 3-step BD-tau**					
	**Kruskal-Wallis** H = 42.940, df = 2, p = 4.74E-10					
	**Dunn's post-hoc**	**group1**	**group2**	**z**	**p_raw**	**p_bonf**
		Control	Chronic-mixed	0.654	0.513	1.000
		Control	Severe-acute	4.760	1.91E-06	5.73E-06
		Chronic-mixed	Severe-acute	5.650	1.65E-08	4.95E-08
	**ANCOVA adjusted for sex and age (using log10-transformation on biomarkers):** F(2,55) = 51.5, p = 2.49E-13					
	contrast	estimate	SE	df	t.ratio	p.value

	Control - (Chronic-mixed)	-0.027	0.111	55	-0.245	0.808
	Control - (Severe-acute)	-0.706	0.106	55	-6.654	2.08E-08
	(Chronic-mixed) - (Severe-acute)	-0.679	0.077	55	-8.826	1.20E-11
**Figure 4A**	**GOSE Outcome (Favorable 5-8 vs. Unfavorable 1-4) differences in 2-step BD-tau**					
	**Two-sided Wilcoxon** W = 44, p = 8.364E-08					
	**Within Severe-acute group:**<br>**Two-sided Wilcoxon** W = 44, p = 2.30E-03<br>**ANCOVA adjusted for sex and age (using log10-transformation on biomarkers)**: F(1,29) = 13.4579, p = 9.76E-04					
**Figure 4B**	**GOSE Outcome (Favorable 5-8 vs. Unfavorable 1-4) differences in 3-step BD-tau**					
	**Two-sided Wilcoxon** W = 51.5, p = 4.63E-07					
	**Within Severe-acute group:**<br>**Two-sided Wilcoxon** W = 51.5, p = 0.0081<br>**ANCOVA adjusted for sex and age (using log10-transformation on biomarkers)**: F(1,28) = 1.852, p = 0.00239					

Table S4. Unadjusted model of performative prediction of Pitt-BD-tau in predicting TBI

Comparison	Measure	N	Thr	AUC	Sens	Spec	Accuracy	PPV	NPV
Control vs Severe-acute	Quanterix BD-Tau	40	2.81	1.000	0.969	1.000	0.975	1.000	0.889
Control vs Severe-acute	2STEP	41	2.80	0.996	0.970	0.875	0.951	0.970	0.875
Control vs Severe-acute	3STEP	40	3.31	0.998	0.969	0.875	0.950	0.969	0.875
Chronic-mixed vs Severe-acute	Quanterix BD-Tau	52	4.33	0.998	0.969	0.950	0.962	0.969	0.950
Chronic-mixed vs Severe-acute	2STEP	54	3.13	0.996	0.970	0.952	0.963	0.970	0.952
Chronic-mixed vs Severe-acute	3STEP	52	4.02	0.988	0.938	0.950	0.942	0.968	0.905

Supplementary Figure

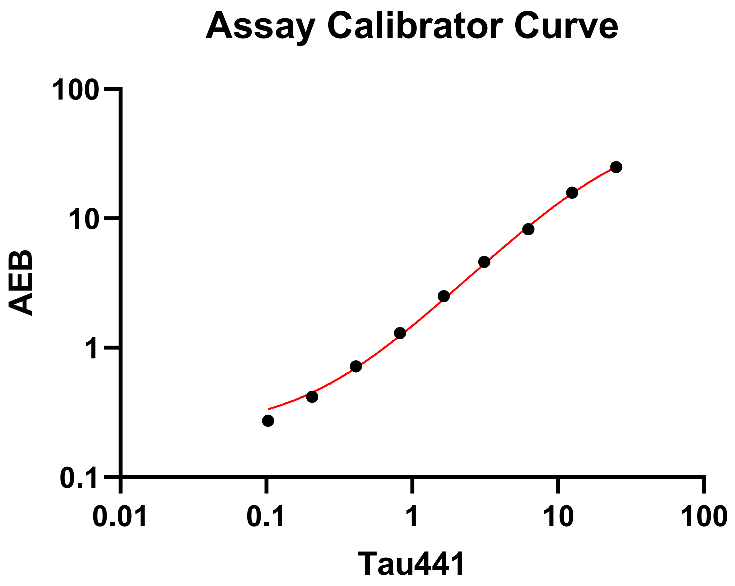

Figure S1: Four-parameter logistic (4PL) regression calibration curve on a log-log scale. The curve was generated with nine non-zero calibrators that are used with the Pitt-BD-tau assay.
